# Reported Mental Health, Diet, and Physical Activity in Young Adult Cancer Survivors

**DOI:** 10.3390/nu15041005

**Published:** 2023-02-17

**Authors:** Acadia W. Buro, Marilyn Stern, Tiffany L. Carson

**Affiliations:** 1 Department of Health Outcomes and Behavior, Moffitt Cancer Center, Tampa, FL 33612, USA; 2 Department of Child and Family Studies, College of Behavioral and Community Sciences, University of South Florida, Tampa, FL 33612, USA

**Keywords:** stress, health behaviors, survivorship, young adult

## Abstract

Young adult (YA) cancer survivors are at increased risk for chronic diseases and face age-dependent stressors that may hinder their ability to maintain healthy lifestyle behaviors. This study examined associations between reported mental health, eating beliefs, and health behaviors in YA cancer survivors. YA cancer survivors aged 18–39 years (n = 225) completed a self-administered REDCap^®^ survey, including the Perceived Stress Scale 10, PROMIS^®^ Anxiety and Depression, Eating Beliefs Questionnaire, National Health and Nutrition Examination Survey Dietary Screener Questionnaire, Godin–Shephard Leisure-Time Physical Activity Questionnaire, and demographic and diagnosis-related questions. Descriptive statistics, bivariate analyses, and multiple linear regression were performed. Participants were mean 31.3 years old and 3.7 years post-treatment; 77.3% were women. Most participants reported White (78%) or Black or African American (11.2%) race and non-Hispanic ethnicity (84%). Adjusting for covariates, perceived stress, anxiety, and depression were associated with increased added sugar intake (*p* < 0.001) and eating beliefs (*p* < 0.001). Perceived stress and depression were associated with reduced vegetable intake (*p* < 0.05). There were no associations with fruit intake or physical activity in the adjusted models. Health behavior interventions for this population may address psychosocial needs by including a stress management or mind–body component. Further research including direct measures of health behaviors is warranted.

## 1. Introduction

Improvements in cancer treatments have led to increased survival rates for young adult (YA) cancer survivors, with an 86% 5-year survival rate for all cancer types [1,2]. This growing cohort of survivors has unique needs for survivorship care [3,4,5] and faces an increased risk of obesity-related chronic health conditions, including diabetes [6,7] and cardiovascular disease [8]. Obesity is also linked to cancer recurrence and mortality for at least thirteen types of cancer [9,10,11,12]. Although the etiology of obesity is multifactorial [13], diet and physical activity are modifiable risk factors. Despite multidisciplinary recommendations for healthy dietary patterns and physical activity levels in YA cancer survivors [14,15], this group shows poor adherence to dietary guidelines [16,17,18,19] and low levels of physical activity [16,20,21]. 

Young adulthood is a critical period to help cancer survivors implement healthy lifestyle behaviors. YA cancer survivors have more unmet psychosocial and behavioral needs [22,23] and tend to be more receptive to physical activity information [24] than older survivors. Emerging adulthood has also been identified as a key transition period for adopting healthy weight control behaviors [25]. Among cancer survivors, this transition period and its related stressors can persist beyond emerging adulthood, with challenges related to late treatment effects, relationships, and changing priorities [26]. Many experience the transition from cancer patient to survivor while also transitioning to independent adulthood [27].

It is known that psychosocial stress can alter diet [28,29] and physical activity [30] behaviors and increase the risk of unhealthy weight gain [31,32] in the general population, but there is a lack of research on associations between psychosocial stress and health behaviors in cancer survivors. To address the gap in the research by investigating the relationship between mental health and lifestyle behaviors in YA cancer survivors, the present study examined associations between reported mental health (i.e., perceived stress, anxiety, and depression) and eating beliefs, dietary intake, and physical activity in a sample of YA cancer survivors. 

## 2. Materials and Methods

### 2.1. Participants and Study Design

This cross-sectional study examined survey data from 225 YA cancer survivors. Eligible participants were (1) diagnosed with cancer, (2) between the ages of 18 and 39 years, and (3) able to read and speak English. Participants were screened for severe cognitive impairments, defined as having a head injury in the past year. No participants were excluded for this reason. Participants were recruited via the Moffitt Cancer Center Adolescent and Young Adult email listserv. Three emails with a link to the REDCap^®^ survey were sent via the listserv over the course of four months. There were 275 participants who started the survey, and 225 who completed the survey and had data available for all variables included in this analysis.

### 2.2. Data Collection

Participants completed a self-administered survey via REDCap^®^, including general demographic and diagnosis-related questions created by the research team and validated measures of perceived stress, anxiety, depression, eating beliefs, dietary intake, and physical activity. Demographic and diagnosis-related characteristics included age, gender, sexual orientation, race, ethnicity, education level, household income, cancer type, time since cancer diagnosis, cancer stage when diagnosed, type of cancer therapy received, time since cancer treatment, and comorbidities.

Perceived stress was assessed by the Perceived Stress Scale 10 (PSS-10) [33], including 10 items with 5-point Likert scale responses to measure the degree to which an individual perceives life events to be stressful. Higher total scores correspond to greater perceived stress. The PSS-10 has been validated in diverse populations and demonstrated to have good internal consistency (α = 0.79) in a large national sample of Americans [34].

Anxiety and depression were assessed by the PROMIS^®^ Anxiety and Depression measures [35,36], 7–8-item instruments with 5-point Likert scale responses that were developed to assess self-reported depression and anxiety. A higher score for each 7-item scale (i.e., depression and anxiety) corresponds to greater symptoms. The instrument has previously been used to screen for emotional distress in cancer survivors [37]. Excellent internal consistency (α ≥ 0.95) has been evidenced in patients with cervical cancer [38]. 

Eating beliefs were assessed by the Eating Beliefs Questionnaire (EBQ-18) [39]. The EBQ-18 includes 18 items with 5-point Likert scale responses that measure the respondent’s number of three types of beliefs related to food and eating that may play a role in binge eating: negative beliefs, positive beliefs, and permissive beliefs. The EBQ-18 has been validated and demonstrated to have good internal consistency (α = 0.88–0.96) in clinical and non-clinical samples [39]. The eating beliefs total score was analyzed for this study, with a higher score reflecting increased severity in eating psychopathology.

Dietary intake was assessed by a 30-item Dietary Screener Questionnaire (DSQ) [40] developed by the Risk Factor Assessment Branch (RFAB) at the National Cancer Institute (NCI) to briefly assess intakes of fruits and vegetables, percentage energy from fat, fiber, added sugars, whole grains, calcium, dairy products, and red and processed meats [40]. The screener is not as accurate as more in-depth methods (e.g., 24 h recall) but can be used to examine interrelationships between diet and other variables. 

Physical activity was assessed by the Godin–Shephard Leisure-Time Physical Activity Questionnaire (GSLTPAQ), a common self-report measure of physical activity that has been validated in healthy adults [41]. The instrument asks participants to report how many times per week they engaged in mild, moderate, and strenuous exercise for 15 min or more. 

### 2.3. Data Analysis

Descriptive statistics were performed for all variables examined. Correlations between independent (i.e., perceived stress, anxiety, and depression) and dependent (i.e., eating beliefs, fruit and vegetable intake, added sugar intake, and physical activity) were conducted. For bivariate analyses that indicated statistically significant associations, stepwise regression with backward elimination was conducted to adjust for sociodemographic characteristics, with a threshold of *p* = 0.15 for removal of variables starting with age, gender (dummy-coded as man vs. woman), race/ethnicity (dummy-coded as White vs. non-White), and income as reported on the demographic questionnaire. The *p*-value for the primary covariate of interest in each analysis was adjusted for type one error inflation using the Holm–Bonferroni procedure. 

## 3. Results

Participants were mean 31.3 years old and 4.7 years post-diagnosis; 77.3% were women. Most participants reported White (78%) or Black or African American (11.2%) race and non-Hispanic ethnicity (84%). The mean score for perceived stress was 22.5 of a maximum 40, and mean T-scores for anxiety and depression were 49.9 and 46.2 of a maximum 82.7 and 81.1, respectively. The mean eating beliefs score was 18.7 of a maximum 90. Mean daily frequencies for added sugar, fruit, and vegetable intake were 2.5, 0.6, and 0.8, respectively. The mean leisure score index for physical activity was 26.6. Descriptive characteristics are summarized in Table 1.

Based on bivariate analyses, perceived stress, anxiety, and depression were positively associated with eating beliefs (*p* < 0.001) and added sugar intake (*p* < 0.05). Perceived stress and anxiety were inversely associated with physical activity (*p* < 0.05), and perceived stress and depression were inversely associated with vegetable intake (*p* < 0.05). Depression was inversely associated with fruit intake (*p* = 0.019). Full correlation results are depicted in Table 2.

Adjusting for covariates, perceived stress, anxiety, and depression were associated with increased added sugar intake (*p* < 0.001) and eating beliefs (*p* < 0.001). Perceived stress and depression were associated with reduced vegetable intake (*p* < 0.01). Perceived stress, anxiety, and depression were not significantly associated with fruit intake or physical activity after adjusting for age, gender, race/ethnicity, and income. In all models, older age was positively associated with fruit and vegetable intake; identifying as a woman was positively associated with vegetable intake and inversely associated with added sugar intake and physical activity; non-White race was inversely associated with vegetable intake; and lower income was inversely associated with fruit intake (*p* < 0.05). Results from multiple regression models are summarized in Table 3.

## 4. Discussion

Self-reported mental health was associated with some eating beliefs and habits in YA cancer survivors. After adjusting for sociodemographic characteristics, perceived stress, anxiety, and depression were associated with increased added sugar intake, and perceived stress and depression were associated with reduced vegetable intake. None of the mental health predictors were associated with physical activity after adjusting for covariates in the adjusted models. 

Although the mean score for physical activity corresponded to an “active” moderate-to-strenuous leisure score index (≥24) [41,42], the mean daily frequency for fruit and vegetable intake was each <1 and mean daily frequency for added sugar was 2.5, indicating poor adherence to American Cancer Society recommendations for healthy dietary patterns [43]. The majority of participants (92.5%) reported elevated stress based on a cutoff PSS-10 score of 13 [34]. Participants in our study also reported high perceived stress compared to adolescent and young adult (AYA) cancer survivors aged 15–35 years (median PSS-10 score of 15) in a previous study [44]. Previous research has found increased levels of distress among YA cancer survivors compared to age-, sex-, and education-matched controls [45]. On the other hand, mean anxiety and depression scores in our study were low compared to existing thresholds that have been previously assessed in YA cancer survivors [46,47].

Mental health variables were not associated with physical activity in the adjusted models. However, perceived stress and anxiety were inversely associated with physical activity in bivariate analyses, indicating that mental health may still be important to address in physical activity interventions for this population. Moreover, identifying as a woman was inversely associated with physical activity in the adjusted models, which is surprising given that women with and without a history of cancer have been reported to engage in more health-conscious behaviors, such as physical activity [48,49]. Physical activity interventions may be particularly important for women cancer survivors in this age group, but further research is needed to understand this finding and the complex relationship between survivorship and physical activity among all YA survivors.

Overall, findings suggested that mental health may be appropriate to address in lifestyle interventions for YA cancer survivors, particularly when targeting added sugar and vegetable intake. Previous research has found associations between perceived stress and energy-dense and/or ultra-processed food intake in young adults from the general population [50,51], and stress management has been shown to mediate this association [50]. Our results also highlighted sociocultural factors to consider in health behavior interventions for YA cancer survivors. Future behavioral interventions for YA cancer survivors may be tailored based on such factors, including participants’ gender, racial/ethnic group, and income level, and dietary interventions in particular may prioritize younger adult survivors (e.g., <30 years).

Limitations of our study include the cross-sectional survey design, small sample size, and self-report instruments (i.e., screeners, short forms). Directionality of associations cannot be established. As this was an exploratory study, we used short screeners rather than including clinical diagnoses or direct measures of dietary intake and physical activity. Although our findings suggest that reported mental health is associated with eating beliefs, added sugar intake, and vegetable intake in YA cancer survivors, additional research is warranted to confirm these findings. Future research is needed that includes larger sample sizes and direct measures of diet, physical activity, and stress (e.g., weighed food records (WFR) [52], accelerometry, and cortisol), as well as clinical diagnoses for anxiety and depression.

## 5. Conclusions

Our findings suggest that future health behavior interventions for this population may address psychosocial needs (e.g., by including a stress management, mindful eating, or other mind–body component). Further research including direct measures is needed to better understand associations between mental health and eating and physical activity habits in YA cancer survivors. 

## Figures and Tables

**Table 1:** Reported mental health, health behaviors, and sociodemographic characteristics in a sample of young adult cancer survivors (n = 225).

Characteristic	n (%)
Perceived stress	Mean ± SD: 22.5 ± 6.2
Anxiety (T-score)	Mean ± SE: 49.9 ± 2.3
Depression (T-score)	Mean ± SD: 46.2 ± 2.8
Eating beliefs	Mean ± SD: 18.7 ± 16.4
Dietary intake ^a^	
Added sugar (daily frequency)	Mean ± SD: 2.50 ± 2.62
Fruit (daily frequency)	Mean ± SD: 0.6 ± 0.6
Vegetables (daily frequency)	Mean ± SD: 0.8 ± 0.7
Physical activity (leisure score index)	Mean ± SD: 26.6 ± 44.5
Age (years)	Mean ± SD: 31.3 ± 5.7
18–29 years	85 (37.8%)
30–39 years	139 (61.8%)
Missing	1 (0.4%)
Gender	
Cisgender man	50 (22.2%)
Cisgender woman	174 (77.3%)
Transgender man	1 (0.4%)
Sexual orientation	
Heterosexual	197 (87.6%)
Homosexual	8 (3.6%)
Bisexual	17 (7.6%)
Asexual	1 (0.4%)
Demisexual	2 (0.9%)
Race ^b^	
Black or African American	24 (11.2%)
White	167 (78.0%)
Asian American or Pacific Islander	14 (6.5%)
Multi-racial	5 (2.3%)
Other ^c^	4 (1.9%)
Ethnicity	
Hispanic or Latino	36 (16.0%)
Not Hispanic or Latino	189 (84.0%)
Highest level of education completed	
Less than high school	1 (0.4%)
High school diploma	21 (9.3%)
Some college	50 (22.2%)
College degree	93 (41.3%)
Some graduate school	13 (5.8%)
Graduate degree	47 (20.9%)
Household income	
Less than $20,000	24 (10.7%)
$20,000 to $34,999	34 (15.1%)
$35,000 to $49,999	29 (12.9%)
$50,000 to $74,999	42 (18.7%)
$75,000 to $99,999	94 (41.8%)
Cancer type	
Bladder	3 (1.3%)
Brain or other nervous system	6 (2.7%)
Breast	36 (16.0%)
Cervical	6 (2.7%)
Colorectal	6 (2.7%)
Head and neck	3 (1.3%)
Kidney	2 (0.9%)
Leukemia	22 (9.8%)
Lymphoma	37 (16.4%)
Liver	2 (0.9%)
Lung	3 (1.3%)
Mesothelioma	1 (0.4%)
Myeloma	2 (0.9%)
Ovarian	4 (1.8%)
Sarcoma	20 (8.9%)
Skin	12 (5.3%)
Testicular	10 (4.4%)
Thyroid	28 (12.4)
Uterine	6 (2.7%)
Vaginal/vulvar	1 (0.4%)
Other ^d^	14 (6.2%)
Missing	1 (0.4%)
Time since cancer diagnosis (years)	Mean ± SD: 4.7 ± 4.6
Cancer stage when diagnosed	
0	40 (17.8%)
I	59 (26.2%)
II	49 (21.8%)
III	43 (19.1%)
IV	33 (14.7%)
V	1 (0.4%)
Type of cancer therapy received ^e^	
Chemotherapy	127 (28.5%)
Radiation	80 (18.0%)
Immunotherapy	43 (9.7%)
Hormonal	32 (7.2%)
Surgery	134 (30.1)
Other ^f^	29 (6.5%)
Time since cancer treatment (years)	Mean ± SD: 3.7 ± 4.0
Comorbidities	
Heart disease	2 (0.9%)
Hypertension	7 (3.1%)
Diabetes	1 (0.4%)
Obesity	22 (9.8%)
Crohn’s disease	3 (1.3%)
Other ^g^	14 (6.2%)
None reported	179 (79.6%)

^a^ Sum of daily candy, doughnuts, cookies, ice cream, soda, sweetened fruit drinks, and sweetened coffee or tea; ^b^ n = 214 due to some participants selecting Hispanic or Latino as their race/ethnicity and some participants selecting more than one race/ethnicity group; ^c^ Haitian, American Indian, Spaniard, and not specified (n = 1 each); ^d^ None specified; ^e^ Some participants received more than one therapy type; ^f^ Autologous and allogeneic transplant, bone marrow transplant (n = 4), chimeric antigen receptor (CAR) T-cell therapy (n = 2), chemoradiation, extracorporeal photopheresis (ECP) and medications for maintenance, natural remedies, narrowband ultraviolet B (NBUVB), none, octreotide/lanreotide, radioactive iodide (n = 3), radioactive iodide, stem cell transplant, targeted therapy (n = 2), tyrosine kinase inhibitor (TKI), topical steroids and creams (n = 1 each unless otherwise stated); ^g^ Anxiety, attention-deficit/hyperactivity disorder (ADHD), complex post-traumatic stress disorder (CPTSD), human immunodeficiency virus (HIV), hypothyroidism, Hashimoto’s disease, kidney disease, liver transplant, second cancer, rheumatoid arthritis (n = 1 each). SD = standard deviation.

**Table 2:** Correlations between perceived stress, anxiety, and depression and eating beliefs, dietary intake, physical activity, and sociodemographic characteristics in a sample of young adult cancer survivors (n = 225).

	Eating Beliefs		Added Sugar Intake		Fruit Intake		Vegetable Intake		Physical Activity	
	r	*p*-Value	r	*p*-Value	r	*p*-Value	r	*p*-Value	r	*p*-Value
Perceived stress	0.317	<0.001 *	0.275	<0.001 *	−0.133	0.046	−0.206	0.002 *	−0.148	0.026 *
Anxiety	0.346	<0.001 *	0.238	0.002 *	−0.070	0.296	−0.130	0.051	−0.162	0.015 *
Depression	0.359	<0.001 *	0.258	<0.001 *	−0.156	0.019 *	−0.172	0.010 *	−0.082	0.222
Older age	0.052	0.434	−0.069	0.379	0.213	0.001 *	0.206	0.002 *	0.075	0.265
Woman	0.091	0.173	−0.192	0.013 *	0.121	0.070	0.199	0.003 *	−0.173	0.010 *
Non-White race	0.131	0.049	−0.024	0.759	−0.040	0.546	−0.160	0.016 *	−0.034	0.612
Lower income	0.104	0.122	0.118	0.130	−0.244	<0.001 *	−0.148	0.027 *	−0.062	0.359

* Significant at an alpha level of 0.05.

**Table 3:** Eating beliefs, dietary intake, and physical activity by perceived stress, anxiety, and depression in a sample of young adult cancer survivors (n = 225).

	Eating Beliefs		Fruit Intake		Vegetable Intake		Added Sugar Intake		Physical Activity	
	β (CI)	*p*-Value	β (CI)	*p*-Value	β (CI)	*p*-Value	β (CI)	*p*-Value	β (CI)	*p*-Value
Perceived stress										
Perceived stress	0.312 (0.182, 0.442)	<0.001 *	−0.078 (−0.208, 0.052)	0.238	−0.188 (−0.314, −0.060)	0.004 *	0.297 (0.146, 0.448)	<0.001 *	−0.128 (−0.263, 0.007)	0.063
Older age	0.078 (0.052, 0.207)	0.237	0.167 (0.037, 0.297)	0.012 *	0.183 (0.057, 0.312)	0.005 *	0.005 (−0.138, 0.147)	0.946	0.076 (0.058, 0.210)	0.265
Woman	0.068 (−0.057, 0.193)	0.286	0.125 (−0.001, 0.252)	0.053	0.209 (0.085, 0.333)	0.001 *	−0.249 (−0.398, −0.100)	0.001 *	−0.173 (−0.304, −0.042)	0.010 *
Non-White race	0.105 (0.022, 0.232)	0.105	−0.014 (−0.145, 0.127)	0.834	−0.168 (−0.293, −0.043)	0.009 *	−0.062 (−0.209, 0.085)	0.408	−0.041 (−0.175, 0.093)	0.546
Lower income	0.026 (−0.109, 0.161)	0.704	−0.203 (−0.336, −0.070)	0.003 *	−0.052 (−0.183, 0.079)	0.433	0.077 (−0.077, 0.085)	0.324	−0.008 (−0.150, 0.134)	0.912
Anxiety										
Anxiety	0.345 (0.216, 0.474)	<0.001 *	−0.031 (−0.161, 0.099)	0.647	−0.122 (−0.253, 0.007)	0.064	0.283 (0.129, 0.437)	<0.001 *	−0.128 (−0.263, 0.007)	0.064
Older age	0.090 (−0.038, 0.218)	0.168	0.169 (0.037, 0.299)	0.011 *	0.183 (0.055, 0.309)	0.005 *	0.029 (−0.122, 0.179)	0.707	0.072 (−0.062, 0.206)	0.291
Woman	0.035 (−0.090, 0.160)	0.580	0.125 (0.003, 0.253)	0.056	0.216 (0.090, 0.342)	<0.001 *	−0.273 (−0.425, −0.121)	<0.001 *	−0.162 (−0.294, −0.030)	0.017 *
Non-White race	0.098 (−0.027, 0.223)	0.125	−0.014 (−0.144, 0.116)	0.832	−0.167 (−0.294, −0.040)	0.010 *	−0.055 (−0.204, 0.094)	0.467	−0.038 (−0.169, 0.093)	0.569
Lower income	−0.034 (−0.165, 0.097)	0.609	−0.216 (−0.349, −0.083)	0.002 *	−0.073 (−0.204, 0.058)	0.272	0.096 (−0.056, 0.248)	0.215	−0.014 (−0.154, 0.126)	0.844
Depression										
Depression	0.349 (0.220, 0.477)	<0.001 *	−0.112 (−0.242, 0.018)	0.093	−0.170 (−0.296, 0.041)	0.009 *	0.289 (0.136, 0.442)	<0.001 *	−0.053 (−0.189, 0.082)	0.443
Older age	0.083 (−0.045, 0.211)	0.203	0.164 (0.034, 0.294)	0.013 *	0.183 (0.055, 0.311)	0.005 *	0.014 (−0.142, 0.170)	0.861	0.080 (−0.055, 0.215)	0.244
Woman	0.060 (−0.065, 0.185)	0.346	0.129 (0.002, 0.256)	0.046	0.211 (0.087, 0.335)	<0.001 *	−0.253 (−0.403, −0.103)	0.001 *	−0.177 (−0.309, −0.045)	0.009 *
Non-White race	0.081 (−0.045, 0.207)	0.205	−0.006 (−0.142, 0.130)	0.930	−0.157 (−0.283, −0.031)	0.015 *	−0.077 (−0.226, 0.072)	0.308	−0.038 (−0.171, 0.094)	0.573
Lower income	0.026 (−0.103, 0.155)	0.692	−0.199 (−0.331, −0.066)	0.003 *	−0.060 (−0.190, 0.069)	0.360	0.086 (−0.067, 0.238)	0.268	−0.027 (−0.166, 0.113)	0.703

Note: CI = 95% confidence interval. * The *p*-value for the primary covariate of interest in each analysis was adjusted for type one error inflation using the Holm–Bonferroni multiple comparison correction method.

## Data Availability

The data analyzed for the current study are available from the corresponding author on reasonable request.
